# “Straight from the heavens into your bucket”: domestic rainwater harvesting as a measure to improve water security in a subarctic indigenous community

**DOI:** 10.1080/22423982.2017.1312223

**Published:** 2017-04-19

**Authors:** Nicholas Mercer, Maura Hanrahan

**Affiliations:** ^a^ Department of Geography and Environmental Management, University of Waterloo, Waterloo, Canada; ^b^ Department of Native American Studies, University of Lethbridge, Lethbridge, Canada

**Keywords:** Indigenous, health, water, security, subarctic, Canada, rainwater harvesting, drinking water, psychological stress

## Abstract

**Background**: Black Tickle-Domino is an extremely water-insecure remote Inuit community in the Canadian subarctic that lacks piped-water. Drinking water consumption in the community is less than a third of the Canadian national average. Water insecurity in the community contributes to adverse health, economic, and social effects and requires urgent action.

**Objectives**: To test the ability of domestic rainwater harvesting (DRWH) for the first time in the subarctic with the goal of improving water access and use in the community.

**Design**: This project utilised quantitative weekly reporting of water collection and use, as well as focus group discussions. DRWH units were installed at seven water-insecure households chosen by the local government. Results were measured over a 6-week period in 2016.

**Results**: Participants harvested 19.07 gallons of rainwater per week. General purpose water consumption increased by 17% and water retrieval efforts declined by 40.92%. Households saved $12.70 CDN per week. Participants reported perceived improvements to psychological health. Because no potable water was collected, drinking water consumption did not increase. The study identified additional water-insecurity impacts.

**Conclusion**: DRWH cannot supply drinking water without proper treatment and filtration; however, it can be a partial remedy to water insecurity in the subarctic. DRWH is appropriately scaled, inexpensive, and participants identified several significant benefits.

## Introduction

This pilot project tested rainwater harvesting in a water-insecure indigenous community of 140 people in Coastal Labrador in subarctic Canada. This paper builds on previous research on water insecurity impacts in the community and identifies domestic rainwater harvesting (DRWH) as a small-scale, inexpensive potential remedy.

Globally, water access is recognised as a human right. In July 2010, the United Nations General Assembly adopted Resolution 64/292, which explicitly “recognised the human right to water and sanitation and acknowledged that clean drinking water and sanitation are essential to the realisation of all human rights” [1]. In 2002, the United Nations Committee on Economic, Social and Cultural Rights adopted “General Comment No. 15”, which defined the right to water as “the right of everyone to sufficient, safe, acceptable and physically accessible and affordable water for personal and domestic uses” [2].

Despite this, access to safe drinking water in many of Canada’s indigenous communities is severely restricted. In 2010, 49 out of 600 First Nations communities surveyed had high-risk drinking water systems, and more than 100 faced ongoing boil water advisories [3]. There are approximately 5000 homes in First Nations communities throughout the country (representing a population of over 20,000 inhabitants) that lack basic water and sewer services [4]. Compared to other Canadians, First Nations’ homes are 90-times more likely to lack running water [5].

Canada has one of the most decentralised water governance systems in the world. The federal government publishes guidelines, but these are not enforceable. Responsibility for water infrastructure funding is devolved to provincial governments. In turn, service delivery, including monitoring, is done by municipal governments [6,7], with their activities and powers defined by provincial governments [8]. Thus, water governance in Canada is characterised by the devolving of responsibilities from senior to junior governments, which frequently lack the necessary resources to ensure water security. As such, small communities with limited means, like Black Tickle-Domino, are vulnerable to water insecurity. Framed another way, Canada’s water system is fragmented due to the involvement of multiple institutions in drinking water management [9]. This fragmentation is the foundation of water insecurity in Canada.

Indigenous communities disproportionately suffer water insecurity in Canada. Water insecurity in remote Indigenous communities “can result in multidimensional consequences including adverse health, economic, social and cultural impacts” [10, pp. 10–11]. For instance, the Federal Provincial and Territorial Advisory Committee on Population Health states that “the incidence of waterborne diseases is several times higher in First Nations communities, than in the general population, in part because of the inadequate or non-existent water treatment systems” [11, p. 102]. Detrimental health effects associated with water insecurity and poor water infrastructure include elevated rates of psychological stress, influenza, whooping cough (pertussis), shigellosis and impetigo [10,12]. Water-washed diseases – infections caused by compromised personal hygiene resulting from inadequate water availability – are also common in many arctic and subarctic communities; examples include trachoma, bacterial skin infections and respiratory infections [13].

Previous research has promoted DRWH as a measure to improve water security in communities with restricted water access [14–17], with its “main advantage [being] to provide water right at the household, suppressing the burden of having to walk long distances to fetch water” [15, p. 1052]. However, most of the existing research has only hypothesised or modelled the potential of DRWH in Africa [15–17]. Very few studies have empirically tested DRWH as water security solution. To our knowledge, DRWH has not been formally tested in Canada’s subarctic Indigenous communities, where water insecurity is particularly acute [3–5].

Water access is severely restricted in the remote Southern Inuit community of Black Tickle-Domino, Labrador, our case study site [18–20]. In common with many remote indigenous communities in Northern Canada, the community lacks a piped water system. Until 2003, when the provincial government installed a potable drinking water unit (PDWU), mainly to service the local fish plant, the people of Black Tickle-Domino relied on shallow ponds, dug wells of just a few feet deep and brooks, such as Porcupine Bay, located as far away as 25 km. Most attempts to construct costly artesian wells have failed. The Local Service District (LSD) operates the PDWU and, because it has no consistent funding, it is forced to charge for drinking water; the actual fee has varied over the years, depending on operating costs and available funding from the provincial government. At present, the PDWU is the only source of treated drinking water in Black Tickle-Domino. It is expensive to operate and utilise (operation and maintenance, direct fees, gasoline, wear and tear on vehicles, etc.); further, it is inconsistently funded by the provincial government and inconveniently located. Finally, it is inappropriately scaled for the community, which contributes to costs, and it is unnecessarily complex and suffers from occasional equipment breakdowns, with parts having to come from as far away as Israel.

As a result, drinking water consumption in Black Tickle-Domino is less than a third of that of the Canadian national average [10]. Our study suggests that the average daily drinking water consumption per capita in the community is approximately 9.2 gallons, and general use water consumption is 71.63 gallons. The average water collection time per household is 51.6 minutes daily. When considering the “access measure guidelines” as established by the World Health Organization (2003), this places the community at a “very high” level of health concern [21]. Furthermore, incomes are low and unemployment is high, which restricts the community’s capacity to improve water security [10]. Thus, the community has a long history of water insecurity, marked by the consumption of unmonitored water as well as significant water access challenges.

Having identified the impacts of water insecurity in previous research, we wanted to take a materialist approach. Working with the community and its umbrella political organisation, we chose DRWH as a possible partial remedy for water insecurity. With two local youths as research assistants, we installed 14 DRWH units, costing $150 each, throughout the community, and observed household participation for 6 weeks (4 July 2016–12 August 2016). We employed a mixed-methods approach in order to assess: (1) changes in drinking/general purpose (e.g. washing) water consumption; (2) perceived changes in health outcomes; and (3) contributions to household economies as a result of the pilot project.

### Study setting

The study took place in the Southern Inuit community of Black Tickle-Domino, which is located on Island of Ponds off the South Coast of Labrador at 53°28′12′′N 55°47′15′′W in the Canadian province of Newfoundland and Labrador. Black Tickle-Domino is part of NunatuKavut (“our ancient land” in Inuktitut) and is a member community of the NunatuKavut Community Council (NCC). The NCC’s comprehensive land claim, filed in 1992, has not yet been accepted for negotiation by the federal government. The people of Black Tickle-Domino and their ancestors practiced seasonal transhumance for hundreds of years, since the Inuit culture was established in Labrador in approximately 1000 CE [22, p. 127]. They wintered in wooded areas in Porcupine Bay and Reeds Pond and travelled to the coast to fish in spring and summer, with Island of Ponds as their main summer station. The Southern Inuit of Black Tickle-Domino were settled on the island year-round in the late 1960s, at the urging of the Roman Catholic Church and the government of Newfoundland, which wanted to end indigenous people’s seasonal movements for the stated purpose of service delivery, especially schooling.

Despite its name, there is no potable water to be obtained on Island of Ponds; there are only shallow ponds containing still water. Extremely glaciated, the island is composed of subarctic tundra with low-lying vegetation and very little soil cover on igneous rock. Fog is common and winds are high, as is snowfall and rainfall. This portion of the Labrador coast receives an average of 189 inches of snow and 209 inches of rain annually, with early summer being the rainiest season [23]. The weather is highly changeable and is heavily influenced by the cold ocean waters of the Labrador Current. Transportation to the island is by plane (although there have been no commercial flights in recent years), coastal boat in summer and autumn and snowmobile or dog team during the long winter. The island is iced-in for approximately 6 months of the year, from December to June.

The people of Black Tickle-Domino are predominantly the descendants of Inuit women and British men who came to Labrador to engage in fishing and trapping. Because of the climate and geophysical environment, the people have always used the social, cultural and economic adaptations of their Inuit, rather than British, ancestors. The Inuktitut language has virtually died out in the community, being replaced by English, although some nouns, mainly related to food, survive. The population of Black Tickle-Domino is currently 140 in 40 households, some of them multi-generational, with a larger seasonal population. The population has declined in recent years with the 2013 closure of the crab processing plant; the population reached approximately 250 people when the plant was in operation. Residents combine government transfer payments with seasonal work outside the community in order to generate household incomes, which are low. Hunting and fishing remain important food sources; as part of their food acquisition efforts, residents pick blackberries and “bakeapples” (cloudberries), hunt ringed and harp seals and Canada geese and fish for cod and salmon. Food sharing is extremely common, in keeping with Inuit cultural values. Many families have small cabins inland where they spend time in winter, accessing fur-bearing animals and water from brooks.

There is an all-grade school with an enrolment of 19 students in 2015–2016 in the community; enrolment will increase due to the presence of 12 pre-school children. Black Tickle-Domino has a medical clinic, staffed by a registered nurse–practitioner and a licensed personal care attendant; the clinic is open 4.5 days a week. In cases of emergency, residents are air-lifted to Goose Bay, the main Labrador service centre, if weather permits. The nearest community, Cartwright, home to approximately 700 Southern Inuit, is about 62 miles away as the crow flies (although it is not possible to travel there so directly); people from Black Tickle-Domino travel to Cartwright via snowmobile in winter and boat in summer. (Cartwright itself is on a gravel road that is a 5-hour drive from Goose Bay.) Thus, Black Tickle-Domino is one of the most remote communities in Newfoundland and Labrador, which is Canada’s most remote province.

## Methods

The research project was designed and conducted in collaboration with two community partners: the NCC, based in Goose Bay, and the Black Tickle-Domino LSD. The LSD is a volunteer local government; under provincial legislation, it cannot collect rates, but it can charge fees for service delivery, such as drinking water purchases. Research was conducted in English, as all community members are fluent in this language.

**Figure 1.**
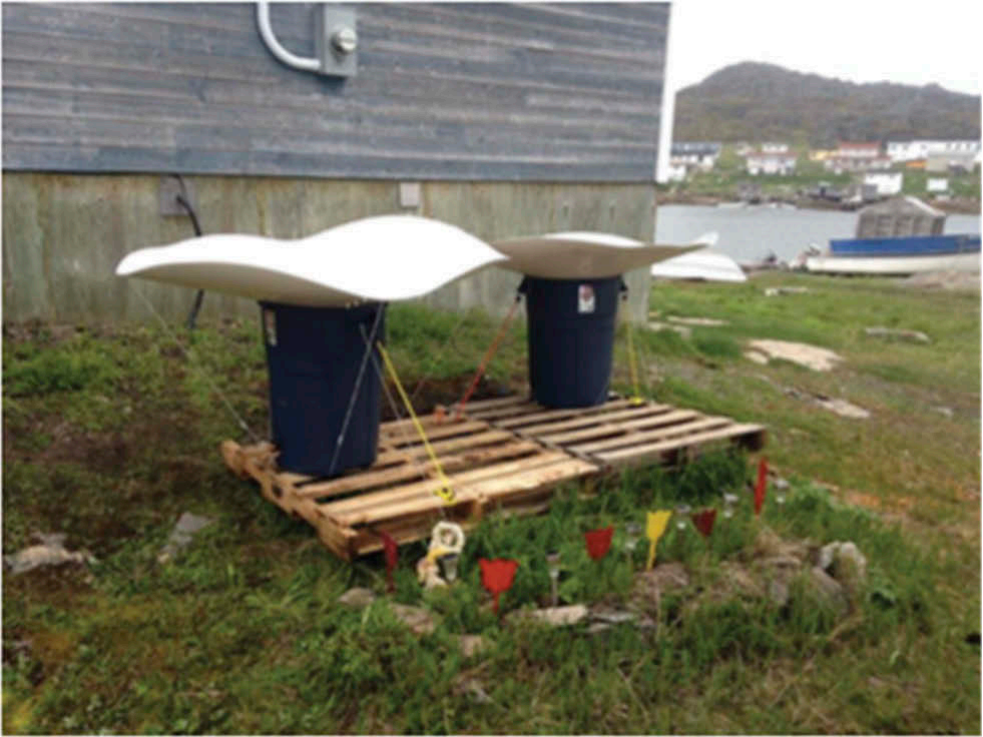
Example of a DRWH unit installed in the community.

We obtained a research grant for our pilot project from the Harris Centre-RBC Water Research and Outreach Fund; this allowed us to purchase, ship and install 14 stand-alone DRWH units in the community (Figure 1). The DRWH units consisted of a 26.4-gallon storage tank, a 59-inch diameter rain saucer attached to the top of the hard rubber storage tank, a basic debris filter and a small spout. We hired two local research assistants, both high school students, to assist with installation and data collection for the 6-week project. A graduate student worked full-time on the project for 14 weeks.

### Research participants

The LSD recruited participants, having had long-term experience with dividing scarce resources in culturally appropriate ways. The LSD recruited seven households, which included 21 individuals; each household received two DRWH units. Inclusion criteria included year- round community residency and direct or indirect participation in water-retrieval efforts, which were chosen in order to help capture water-retrieval experiences. Reflecting the Inuit values of social equality and inclusion, the LSD selected households that were highly water insecure.

### Data collection

We used a mixed-methods approach consisting of a self-administered survey and focus group discussions. The self-administered pre-project survey posed “baseline questions” that allowed us to gather information on household hours dedicated to water collection, water collection costs (direct fees, fuel, maintenance, wear and tear on vehicles, etc.) and household water consumption (both general purpose and drinking water) prior to the pilot project. Working with the graduate student and the local research assistants, household participants updated their surveys weekly throughout the pilot project and recorded the amount of rainwater harvested.

We held focus group discussions at the beginning and conclusion of the pilot project. The introductory focus group discussion (n=5) contained open-ended questions relating to current household water consumption, water retrieval efforts, barriers to water access and pilot project expectations. The concluding focus group discussion (n=5) contained open-ended questions relating to the pilot project results, DRWH unit effectiveness and general feedback. The questions allowed for probing on project impacts. Due to the small sample size, we organised focus group discussions in order to collect qualitative data related to the quantitative survey data. The (Institutional) Research Ethics Board, as well as the NCC Research Advisory Committee, approved this research.

### Data analysis

We used basic descriptive statistics in the quantitative surveys. We used Excel version 15.13.1 software to organise, manage and analyse the survey data. For qualitative data, we used content analysis, applied to focus group transcripts, which were transcribed by a professional academic service [24]. We read the transcripts and inductively built an initial codebook. We reviewed all of the transcripts in order to ensure that the codes comprehensively encompassed key themes. We then used NIVO version 11.1.1 qualitative analytic software to organise, manage and analyse the qualitative data. In order to enhance the credibility of the project [25], we prepared a “preliminary findings” document for our community partners and project participants, which allowed for the giving of feedback. This document later became the basis of the project’s results analysis.

## Results and discussion

### Self-reported data

Table 1 provides an overview of the data collected relating to water retrieval and consumption. Baseline estimates are also reported.

**Table 1.** Impacts of DRWH on water access and consumption.

Weekly attribute	Pre-project (baseline)	Pilot project results	Percentage change
Household hours spent collecting water	6.06	3.58	−40.92%
Household dollars spent collecting water	$30.42	$17.72	−41.75%
Household general purpose water consumption	214.9 gallons	251.65 gallons	17.10%
Household drinking water consumption	27.6 gallons	26.12 gallons	−5.36%
Amount of rainwater harvested	N/A	19.07 gallons	N/A

### Positive impacts of the project

#### Effectiveness of DRWH units

DRWH has been utilised in developing countries, especially on the African continent, where it has been favourably viewed as a response to water access difficulties [15–17]. Until now, DRWH has been tested only in warm to hot climates and not in a climate as harsh as that of subarctic Canada. Climate – especially snow storms, severe cold and precipitation causing damage to unpaved roads – is a barrier to water access in Black Tickle-Domino. Our results demonstrate that DRWH can be an effective method for increasing water access in water-insecure communities in the Canadian subarctic. On average, household participants reported harvesting 19.07 gallons of rainwater per week (Table 1), or 8.9% of baseline general purpose water consumption. The project was received well in the community; as one participant said, “The project itself was amazing, I found that my system collected water really well.”

As discussed with participants, only small-scale DRWH units were utilised because of budget constraints; units with larger surface areas (such as rooftop rainwater harvesting) would be capable of harvesting greater volumes [26]. Research suggests that 1 cm of rain on 100 m^2^ of roof yields 10,000 l (2641.7 gallons) of water [27]. Average historical monthly rainfall in Black Tickle-Domino from June to October is 7.18 cm [28], suggesting that a 100-m^2^ rooftop would yield approximately 19,000 gallons of rainwater monthly; this is significant, considering that current monthly general purpose water consumption is only 930.52 gallons in the community. Possibilities associated with other methods of DRWH, such as rooftop rainwater harvesting or the use of larger rain saucers, may be pursued by the community and the NCC.

#### Increased general purpose water consumption: increased personal hygiene

Participants were able to increase their general purpose water consumption; general purpose water is used for personal hygiene, washing dishes and clothes and household cleanliness. Baseline household consumption was 214.9 gallons/week and increased to 251.65 gallons/week throughout the project, an increase of 17.10% (Table 1). Because water consumption is so restricted in the community, participants saw this increase as significant. According to one participant, “You have that much more water you can use [as a result of DRWH], because it is perfect water for general use.” As Black Tickle-Domino residents are aware, restricted water access compromises personal hygiene, and poor personal hygiene contributes to adverse health effects [12,13,29]. Participants reported that DRWH made them feel more comfortable using water for personal hygiene and general cleanliness. In addition, they favoured rainwater over PDWU water because of rainwater’s aesthetic appeal; PDWU is frequently discoloured, partially the result of its high iron content, with a “yellowish cast” that is a disincentive to using it for personal hygiene and household cleaning. As stated by one participant, “[A main benefit of DRWH] was washing your dishes and your clothes; [the water] was really clean and it smelled good, before [PDWU water] smelled like apple juice … at least we can [now] wash a good shirt and it comes out white.” Another participant added, “[A main benefit of DRWH] was your own general washing purposes. Better than the old scuzzy [PDWU] water.”

#### Financial savings

As Figure 1 demonstrates, in some cases, participants accrued substantial financial savings. Pre-project, participating households reported spending $30.42 CDN weekly on water retrieval efforts. Throughout the pilot project, participating households reported spending $17.72 CDN weekly on average, suggesting weekly savings of $12.70 CDN as a result of the project. The average yearly income in the community is $11,068 CDN, and water retrieval is a severe strain on household financial resources [18]; the costs of water retrieval are part of a web linking poverty, water security and food security. Therefore, even small financial savings may have wide-ranging social and economic benefits, increasing purchasing power, which allows people to purchase more food, for instance. Individual and community empowerment cannot be discounted either, as we discuss below.

Financial savings resulted from decreased water retrieval efforts, mainly in terms of fewer dollars spent paying others in the community to collect water for them, fewer dollars spent in direct fees to the PDWU and fewer dollars spent on fuel for and wear and tear on vehicles used to retrieve water. As one participant stated, “Instead of paying somebody twice a week to get water, I was only paying once a week – so [over the 6-week pilot project] I saved $120.” Another participant stated, “[Every rainwater barrel harvested] was one less trip I had to go way in there [to the PDWU]. The wear and tear savings were more than the actual money [fees at the PDWU], because you have to go all the way in across bedrock and bumps, then all the way back.”

#### Decreased water retrieval efforts

DRWH is a good tool for decreasing physically strenuous water-retrieval efforts [15]. Unlike as in many developing countries, water retrieval is a male chore in Black Tickle-Domino, which is a heavily gendered community derived from its hunting culture and history. Due to the physical demands of water retrieval followed by carrying water into their houses, virtually every man in Black Tickle-Domino has chronic pain due to muscular–skeletal injuries [10]. Some men in the community feel that they cannot leave the island for treatment as their families are dependent on them for food acquisition and water retrieval; therefore, they live with injuries and pain. The pilot project suggests that DRWH is a viable method for decreasing water retrieval efforts in water-insecure communities; weekly household water retrieval hours decreased from 6.06 to 3.58 hours, a decrease of 40.92% (Table 1). Participants explained that by harvesting general purpose water from their DRWH, they were able to limit the number of weekly trips to the PDWU, which meant less lifting and carrying of heavy water and, potentially, fewer injuries.

#### Decline in psychological stress: optimism related to the project

Although this was a small-scale project, one of our most significant findings was its impact on mental health in Black Tickle-Domino, which was identified as a pressing issue in previous research [18–20]. Water insecurity is associated with high levels of psychological stress [10]; in Black Tickle-Domino, water access is the central cause of water insecurity-related stress, such as during winter storms when people are confined to their homes and cannot retrieve water or when the shallow wells dry up, as they do on occasion.

Most participants in this study reported declines in psychological stress related to water insecurity. One participant said, “[Before the project] I have been down in my house for two and three days sometimes with no water at all,” later adding, “[DRWH] is security.” Another participant added, “[Due to DRWH] you do not have to be so conservative [with your water].” Thus, participants were able to rely less on the traditional coping mechanisms of extreme water conservation, water hoarding and doing without water. Even a small degree of improved access was linked to a decline in participants’ stress levels. General feelings of “optimism” contribute to positive mental health outcomes [30,31]. Participants expressed optimism and positive emotional benefits as a result of the project, which may contribute to further improvements in mental health. As stated by one participant, “I like the look of it [their unit]. I am a really proud [pilot project] participant, I love it.” Another participant added, “Thank you for trying it [DRWH] and opening our eyes … it makes you as a person explore different options [for water security].” Thus, DRWH was associated with a renewed sense of empowerment regarding a problem that has long been viewed as intractable.

### Project limitations

#### No increases in drinking water consumption

An objective of the pilot project was to increase household drinking water consumption; we expected that participants would have more available general purpose water, which would allow them to increase their efforts to retrieve drinking water. As explained to participants, the water harvested from the DRWH units was untreated, unmonitored and unsuitable for ingestion; however, by providing some general purpose water, we hoped that participating households could redirect some of their water-retrieval efforts to retrieving drinking water.

With proper treatment and filtration, DRWH may have positive impacts on drinking water consumption; however, treatment and filtration were not feasible for this pilot project due to time and budget constraints. As such, DRWH did not appear to have a positive impact on drinking water consumption. The baseline household drinking water consumption was reported as 27.6 gallons per week; this figure remained stable throughout the project (Table 1). As stated by one participant, “It [DRWH] would not change the water consumed, because you could not drink it.” Participants chose to take “a break” from water retrieval rather than redirect their efforts. This speaks to the strenuous efforts necessary for water retrieval.

#### Difficulty retrieving water from units

Participants reported that it was difficult and physically strenuous to harvest water collected in the DRWH unit when using the spout and buckets: “I could not get the bucket under the nozzle”; “I think it [removing water from the DRWH] would be more work than actually going up to the water treatment plant [PDWU].” Participants enthusiastically identified potential improvements to the technology we used; these included inexpensive garden hoses that could bring water directly into houses, and better placement of the units to make pouring easier.

#### Wind being a factor in system placement

Occasionally, strong wind speeds, which occurred for a total of 3–4 days during the project, posed challenges to DRWH. Units tipped over or came apart due to high winds, and water was lost. As stated by one participant, “… the wind we get around here, it [the rain saucer] blew off a couple times.” Most participants suggested that over the course of the 6-week pilot project, the wind was a limitation, but not an insurmountable challenge. Units needed to be sheltered from winds so that they were secure. We used bungee cords to tie them to wooden pallets fixed to the ground, but, at times, this was not sufficient. The key to remedying this is strategic unit placement based on local experience and knowledge of weather conditions and related factors.

### Additional impacts of water insecurity

An unexpected positive result was that the pilot project built on existing literature on water insecurity in Black Tickle-Domino and elsewhere by identifying additional dimensions of water insecurity, mainly through the focus group discussions.

#### Decreases in recreational activity and leisure

Recreational activity and leisure have positive implications for physical, emotional, social and cognitive health [32–34]. In Black Tickle-Domino, strenuous water retrieval efforts and their impacts on the community’s infrastructure, specifically its unpaved road, appear to contribute to decreases in recreational activity and leisure, which may have adverse health implications. As stated by one participant, “Every evening a few years ago, when the road [used for water retrieval] was not as bad, my husband and I would go out in the evening and go for a [all-terrain vehicle] ride; but you cannot do that now. There is no joy in joyriding.” Another participant added, “[DRWH] could give people a break. They would not have to do so much [water retrieval], they are older people, so they would be able to stay home and relax more.” Because of their lived experience, the links between water insecurity, mental health and recreation as a remedy were well understood by participants. Participants repeatedly expressed a desire for some relief from the relentless difficulties of life in a water-insecure community.

#### Impacts for food security

Water insecurity strains households, forcing people to knowingly make cheaper, unhealthy food choices [10]. In Black Tickle-Domino, parents cope with this by consuming junk food and giving it to their children because they cannot afford healthier food choices; this happens with knowledge and regret, and is seen as an undesirable but necessary choice [10]. Participants in this project and in previous research projects readily identified the connections between water and food security [10]. With reference to food and water security, one DRWH project participant stated that “one does not come without the other.” This project identified another negative food-related impact: a disincentive to trying vegetable gardening, which would require substantial amounts of water. Root crops, such as potatoes and carrots, are incorporated into the local diet, especially during the Sunday dinner ritual. These have to be purchased, however, and can be expensive and in poor condition due to transportation over long distances. Despite limited soil, residents would like to include home-grown vegetables as part of their food acquisition strategies, but water access limits this possibility.

#### Social “inequality” based on water access

Social inequality leads to adverse health impacts such as feelings of hopelessness [35,36], and runs counter to Inuit values. In Black Tickle-Domino, different household levels of water access contribute to feelings of “inequality” or social stratification, which may have adverse psychological health outcomes. A small number of community members have working artesian wells, some have vehicles to help with water retrieval and some use long outdoor hoses in order to run water to their homes, while others lack these amenities and are more water insecure as a result. As one participant stated, “I take care of two seniors, and they got hoses running up the road [to a water source], so they [essentially] have running water.” Another participant immediately added, “I wish I had running water,” followed by another who stated, “Me too.” The pervasive sense of social inequality adds to people’s stress levels. The choice of the LSD to recruit extremely water-insecure householders to the project reflects both awareness of and concern for this inequality and its impacts.

#### Feelings of fear: water retrieval and wildlife

General fear and anxiety have adverse implications for mental health [37,38]. A significant source of fear and anxiety in Black Tickle-Domino is the threat of wildlife (particularly polar bears) during water retrieval. Participants report that, in recent years, there has been an increase in polar bear appearances in and around the community, possibly due to climate change. One researcher witnessed three bears in Black Tickle-Domino in the spring of 2013, which necessitated gun carrying, as polar bears are dangerous animals. Children are kept indoors during this time, and anyone venturing outdoors must be protected by someone who is skilled in shooting; in a gendered community, this restricts the movement of women. Women also worry about their male family members who are responsible for travelling to the PDWU or elsewhere to collect water: “When we have polar bears every evening, lingering around, I do not let my husband go [retrieve water] by himself, because of the danger. He could be bending over doing something, and they [polar bears] can come right around. You almost need somebody to watch your back, somebody to go with you. So that danger is right there and lurking.”

DRWH can partly alleviate this problem, as it is associated with fewer water-retrieval trips and people can retrieve water from units near their homes, which offer shelter in case polar bears appear.

## Conclusion

Urgent action is required for the many remote indigenous communities in Canada that experience persistent water insecurity; access to safe drinking water is a human right and a major human health issue with physical and emotional dimensions. Through its commitment to a fragmented water governance system marked by the devolving of responsibilities without adequate resources, Canada continues to neglect the right to safe, affordable and accessible drinking water. Water insecurity is particularly acute in remote places such as Black Tickle-Domino that lack piped water. The impacts of water insecurity are wide ranging and inter-related, including financial, social and health impacts.

This project is one of the few to test DRWH globally and the first to test DRWH in the subarctic. In Black Tickle-Domino, DRWH led to increased general purpose water consumption, perceived improvements in psychological health, decreased water-retrieval efforts and household savings. Although it provided only partial relief and did not lead to improved access to potable drinking water, participants reported positive feelings overall about the project.

Without technical alteration, DRWH cannot meet drinking water needs, but DRWH unit limitations can be mitigated through measures such as careful unit placement in response to winds. While recognising the small scale of this pilot project in subarctic Canada, DRWH emerges as a potential partial remedy, one that is appropriately scaled and inexpensive. This is particularly true if DRWH were combined with other water access methods and if enhancements were used; these might be small scale, such as inexpensive garden hoses, or more elaborate, enabling rooftop water collection, for instance. We encourage further research across the subarctic in order to address pressing water insecurity problems and their impacts on human health.
